# Wearable Sensors Applied in Movement Analysis

**DOI:** 10.3390/s22218239

**Published:** 2022-10-27

**Authors:** Fabien Buisseret, Frédéric Dierick, Liesbet Van der Perre

**Affiliations:** 1 Centre de Recherche, d’Étude et de Formation Continue de la Haute Ecole Louvain en Hainaut (CeREF Technique), Chaussée de Binche 159, 7000 Mons, Belgium; 2 Service de Physique Nucléaire et Subnucléaire, Research Institute for Complex Systems, UMONS Université de Mons, Place du Parc 20, 7000 Mons, Belgium; 3 Centre National de Rééducation Fonctionnelle et de Réadaptation–Rehazenter, Laboratoire d’Analyse du Mouvement et de la Posture (LAMP), Rue André Vésale 1, 2674 Luxembourg, Luxembourg; 4 Faculté des Sciences de la Motricité, UCLouvain, Place Pierre de Coubertin 1-2, 1348 Ottignies-Louvain-la-Neuve, Belgium; 5 DraMCo Lab of the Electrical Engineering Department, KU Leuven, 9000 Ghent, Belgium

Recent advances in the miniaturization of electronics have resulted in sensors whose sizes and weights are such that they can be attached to living systems without interfering with their natural movements and behaviors. They may be worn on the body as accessories or as part of clothing, enabling personalized mobile information processing. Wearable sensors enable unobtrusive and continuous monitoring of body orientation, movements, and various physiological parameters during real-life activities. Thus, they may become crucial tools not only for researchers but also for clinicians, as they have the potential to improve diagnosis, better monitor disease progression, and thus individualize treatment. We also expect that after the SARS-CoV-2 crisis, interest in devices that promote telemedicine, such as low-cost wearable sensors, will increase significantly.

To be used in real-life situations, wearable sensors should meet the following three criteria: (1) *Be imperceptible to the wearer*. They should have wireless connectivity and consume little power. An example of algorithm development that optimizes both gesture recognition and energy consumption is presented in [1]. There, a finger gesture recognition system was developed using a lightweight multi-layer perceptron implemented on a low-end micro-controller unit with a two-axis flex sensor. The final prototype achieves up to 95.5% recognition accuracy while consuming less than 2.74 mJ of energy per gesture on a low-end embedded wearable device, which is 10% better than previous algorithms. (2) *Be intuitive to install*. The developed systems should provide high-performance body fixation solutions that are easily accepted by the user. Moreover, the electronic system should be self-calibrating and operating. An interesting way to increase the acceptance in domestic applications may be to use smartphones—very broadly accepted electronic devices—as control devices for the developed sensors. In [2], it is shown that dystonia assessment using smartphone-coupled inertial sensors and machine learning is a promising way to detect dystonia in real-life applications. (3) *Provide accurate and easy-to-interpret information*. Cross-platform interfaces that enable secure data storage and easy data analysis and visualization are needed. As an illustration, using Inertial Measurement Units (IMUs) to assess gait pattern evolutions during a 6-min walk test before and after a supervised exercise training program, the authors of [3] obtained such easy-to-interpret information in patients with symptomatic peripheral artery disease of the lower extremities. Two results can be quoted: a significant increase in walking speed after supervised exercise training and a significant positive correlation between the change in stride length and the change in 6-min walking distance. Therefore, the use of IMUs with the aim to investigate gait pattern during physical examination has potential applications for optimizing exercise prescription in patients with peripheral artery disease. Beyond the above examples, the papers published in this Special Issue show that several domains may benefit from wearable sensors when these three criteria are considered. 

Sport is a clear example of a domain where imperceptible sensors are needed so as not to interfere with movement. The information from the sensors may help to improve the efficiency of training through accurate biofeedback. The authors of [4] have shown that a system of eight IMUs was able to identify the different phases (stance times) in a 1000 m speed skating trial for 12 competitive athletes. The IMUs results compare well with a foot pressure detector, which is considered the gold standard: between 90.1% and 96.1% for the average stance time. In [5], it is shown that two IMUs attached to the ski boots of nineteen experienced alpine skiers allow researchers to distinguish between an experienced skier and a beginner by comparing the recorded time series with those of a group of reference skiers. More generally, wearable sensors offer accurate methods of monitoring real-time movement parameters during sport, with an expected high relevance in optimizing training programs and performance or in minimizing risk of injury [6,7]. Note that wearable sensors also offer non-invasive and portable techniques to monitor the sports practices of persons with disability, especially in wheelchair sports [8].

Wearable sensors may also provide clinicians with additional quantitative information when assessing musculoskeletal conditions, such as neck pain and low-back pain. Regarding neck pain, the authors of [9] used a single IMU placed on a participant’s forehead while performing a test to assess sensorimotor performance of the neck through repeated head rotations. A Linear Support Vector Machine can discriminate acute and subacute non-specific neck pain patients from healthy control participants with 82% accuracy by analyzing time series of angular speed and acceleration. The study was conducted with 38 acute and subacute non-specific neck pain patients and 42 healthy control participants and demonstrates that machine-learning methods can provide relevant information from relatively small datasets. The same observation is made in [10], where the kinematics of 20 patients with chronic low-back pain (CLBP) and 20 healthy participants without CLBP were recorded from three IMUs attached to the participants while they performed 1-min repetitive bending (flexion) and return (extension) trunk movements. It was found that Gaussian Naive Bayes machine learning achieved 79% accuracy in identifying CLBP patients. Moreover, machine learning identified that simple kinematic indicators were sensitive to low-back pain and therefore could gradually be used by clinicians in the assessment of CLBP patients. Machine learning can even go beyond binary classification in CLBP patients, as shown in [11]. From the video analysis of 115 CLBP participants lifting an 8 kg weight, Ward clustering suggests that there are four different lifting techniques in people with CLBP. One of the clusters, moving the trunk the least and the knee the most, demonstrates the least pain self-efficacy. Again, these results may help clinicians determine the best motor strategies to relieve pain in their patients. 

A final topic explored in this Special Issue is gait analysis and its relationship to fall risk in the elderly. One challenge in this population is the implementation of automated gait assessment for continuous monitoring, either at home or in care institutions and hospitals. In [12], an IMU was used to assess patients with automated assessment based on the Berg balance scale. Optimal agreement (98.4%) with the therapist’s scoring can be achieved using a one-dimensional convolutional neural network and a gated recurrent unit in a population of 53 hospitalized patients with brain diseases aged 50 to 80 years. Finally, it was shown in [13] that additional information from a single IMU, placed on the lower back of 73 care institute residents who performed a Timed-Up and Go (TUG) test considerably improved fall risk prediction. Kinematic observations and TUG time were included in a multiple logistic regression. The proposed new test, called i+TUG, achieved an accuracy of 74.0%, with a specificity of 95.9% and a sensitivity of 29.2% in classifying residents into fallers and non-fallers. 

Beyond applications in elderly aiming at favoring an autonomous, active, and healthy ageing [14], wearable sensors may bring important improvement in monitoring patients with neurological diseases. As shown in the review [15], e-health approaches, including wearable sensors, may be beneficial for self-management and disease understanding of patients suffering from multiple sclerosis. Wearable motion sensors can be helpful in measuring physical activity of patients suffering from multiple sclerosis [16]. Another case of interest is the application of motion sensors to detect freezing of gait (FOG) in Parkinsons’s disease, i.e., a gait disturbance typical of the mid- and late-stages of the disease. As discussed in the review [17], many challenges are still to be addressed in FOG detection, such as building large enough datasets allowing a more accurate detection via machine-learning techniques. In addition, wearable sensors may be used to estimate the metabolic energy expenditure and physical activity levels of different intensities in stroke patients with hemiparesis [18].

Wearable sensors can clearly bring great value in the analysis of movement, in sports as well as medical contexts, and not in the least for patients suffering from chronic diseases. While the potential is shown in the papers presented here and many others, we are confident that with further development of hardware and signal processing, many new opportunities will follow.
